# Reduction of Blood Loss by Intra‐articular Injection of Tranexamic Acid Combined with Knee and Hip Flexion at 45° During Primary Total Knee Arthroplasty: A Randomized Controlled Trial

**DOI:** 10.1111/os.12814

**Published:** 2020-10-22

**Authors:** Jian‐qi Yang, Lin Yang, Jian‐shao Tan, Kun‐ping Huo, Liang Zhao, Dao‐zhang Cai

**Affiliations:** ^1^ Department of Orthopaedics The Third Affiliated Hospital of Southern Medical University Guangzhou China; ^2^ Department of Orthopaedics The First People's Hospital of Foshan Foshan China; ^3^ Hebei Medical University Shijiazhuang China

**Keywords:** Blood loss, Extension, Flexion, Leg position, Total knee arthroplasty, Tranexamic acid

## Abstract

**Objective:**

To explore the hemostatic effect of intra‐articular administration of tranexamic acid (TXA) combined with knee flexion in total knee arthroplasty (TKA).

**Methods:**

This randomized controlled trial was conducted at the Third Affiliated Hospital of Southern Medical University (Guangzhou, China) from January 2017 to February 2018. The patients were randomized 1:1 to the TXA group (TXA 500 mg into the joint after closure, knee, and hip flexed at 45° for 4 h) or the control group (physiological saline, with limb fully extended). The primary endpoint was postoperative hemoglobin reduction. The postoperative levels of hemoglobin were measured at four time points: 6 h after operation, and on the first, second, and third postoperative days. Calculated blood loss (CBL) at 3 days, transfusion rate, range of motion (ROM), VAS pain score, and knee circumference increment were the secondary endpoints. Ninety‐four (47/group) patients were analyzed.

**Results:**

Postoperatively, there were statistically significant differences between the TXA and control groups in CBL (791 ± 212 mL *vs* 1175 ± 273 mL, *P* < 0.05). Hemoglobin reduction was significantly lower in the TXA group (2.0 ± 0.9 g/dL *vs* 4.5 ± 0.7 g/dL, *P* < 0.05). Based on the transfusion criteria, 3 out of 47 (6.4%) patients in the TXA group and 13 out of 47 (27.6%) patients in the control group received blood transfusions (*P* = 0.006). ROM (90.8° ± 6.2° *vs* 87.6° ± 6.4°, *P* = 0.004), VAS pain score (4.1 ± 1.1 *vs* 4.8 ± 1.3, *P* = 0.004), and KCI (2.4 ± 0.9 cm *vs* 3.2 ± 1.0 cm, *P* = 0.01) were better in the TXA group compared with thecontrols. There was no deep venous thrombosis (DVT), wound infection or other adverse events in either group. In the control group, 2 patients had a fever after blood transfusion.

**Conclusion:**

Intra‐articular injection of TXA combined with knee and hip flexion at 45° can effectively attenuate CBL and hemoglobin reduction during primary TKA, without an additional adverse event.

## Graphical abstract

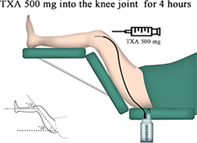

## Introduction

Osteoarthritis (OA) of the knee is characterized by articular cartilage loss, bone remodeling, and periarticular muscle weakness, resulting in knee joint pain, swelling, deformity, and instability^1^. The estimated global prevalence of knee OA in 2010 was 3.8%^2^. The Osteoarthritis Research Society International (OARSI) states that patients with knee osteoarthritis (OA) not achieving adequate pain relief and functional improvement from both non‐pharmacologic and pharmacologic treatment should be considered for joint replacement surgery^3^. Total knee arthroplasty (TKA), followed by nonsurgical treatment, improves pain, symptoms, and quality of life, but serious adverse events are more common with TKA than with conservative treatments^3^.

Although there has been tremendous progress in surgical techniques in recent decades, unexpected and excessive blood loss during TKA remains an issue. Indeed, the total blood loss can reach 1426 mL on average after TKA^4^, leading to the need for blood transfusion in 53.5% of patients^5^ and posing the risk of blood transfusion‐related complications in the perioperative period^6^, including disease transmission, hemolytic reaction, fluid overload, impaired hemodynamics, acute lung injury, coagulation diseases, allergic reactions, and febrile reactions. These complications can result in poor patient outcomes, such as longer hospitalization, higher costs, and even death^7^, ^8^, ^9^, ^10^. The allogeneic transfusion is associated with decreased immunity and increased risk of prosthetic infection^11^, ^12^. Many methods have been proposed to avoid excessive blood loss and transfusion‐related complications, including autologous blood transfusion^5^, ^13^, cryotherapy^14^, hypotensive anesthesia, and pressure bandaging, with some success. Nevertheless, efficacy is limited, and some of these methods, such as autologous blood transfusion, are expensive. Therefore, there is still a clinical need for simple, safe, and effective ways to reduce blood loss for patients undergoing primary TKA. The aims of blood conservation strategies are to avoid allogeneic transfusion and to maximize hemoglobin levels in the postoperative period, leading to better outcomes and lower costs^7^. The strategies should be individualized^7^, ^15^, ^16^. The preoperative strategies include correction of anemia, maximization of the red blood cell mass, stopping anticoagulants, and antiplatelet drugs (but this has to be carefully weighed with the risk of cardiovascular events), and preoperative autologous blood donations^7^. The intraoperative strategies include careful planning and surgical techniques, normovolemic hemodilution, perioperative red cell salvage, and the use of topical fibrin sealants^7^. The postoperative strategies include reinfusion drains and the use of antifibrinolytic agents^7^. The various strategies available can be inconvenient in terms of preparation, costs, and risks of complications. Nevertheless, among them, the postoperative use of antifibrinolytic agents is promising^7^.

Tranexamic acid (TXA) is a synthetic derivative of lysine that exerts an antifibrinolytic effect. Since it was first used in 1995 to enhance hemostasis and to reduce blood loss during TKA^17^, the administration of TXA during TKA has received extensive attention in the management of blood loss during orthopaedic surgeries. The most popular routes of administration of TXA include intravenous administration and injection into the local joint cavity. TXA administration^18^, ^19^, ^20^ and limb positioning^21^, ^22^, ^23^ are simple methods that have been widely used to control blood loss in TKA. The results are generally positive, but the results among some individual studies are conflicting^24^, ^25^, ^26^.

Even if the hemostatic effects of TXA and limb positioning are specific, pursuing a better hemostatic effect is necessary to avoid adverse consequences during TKA and to provide patients a faster recovery. To our knowledge, only a few studies have reported the combined effect of these two methods. Therefore, the aims of the present study were: (i) to examine the effects of the combination of TXA administration and joint flexion for hemostasis on blood loss and transfusion requirements in patients undergoing TKA; (ii) to examine the range of motion (ROM), the visual analog scale (VAS) pain score, and the knee circumference increment (KCI); and (iii) to examine the occurrence of complications.

## Methods

### *Inclusion and Exclusion Criteria*

The inclusion criteria were: (i) diagnosis of moderate or severe knee OA (grade 3 or 4, based on the degree of erosion);^27^ and (ii) ineffective conservative treatment before undergoing primary TKA.

The exclusion criteria were: (i) <55 years of age; (ii) any hemorrhagic blood disease; (iii) hemoglobin levels <10 g/dL; (iv) history of thromboembolic disease; (v) rheumatoid arthritis; (vi) traumatic arthritis; (vii) any disorder of the hips; (viii) preoperative use of anticoagulants; or (ix) TXA contraindications.

### *Study*

This randomized controlled trial was conducted at the Department of Orthopedics of the Third Affiliated Hospital of Southern Medical University (Guangzhou, China) from January 2017 to February 2018. This trial was approved by the ethics committee of the Third Affiliated Hospital of Southern Medical University. Written informed consent was provided by all participants. Trial registration: ChiCTR‐INR‐16010287, registered at chictr.org.cn.

### *Randomization*

The patients were randomized 1:1 to the TXA and control groups equally by a third‐party junior doctor who opened sequentially numbered sealed opaque envelopes that were initially prepared by an independent statistician.

### *Blinding*

Because of the nature of the intervention, the patients, investigators, and nurses could not be blinded to grouping. The postoperative assessments were performed by a surgeon who was blinded to grouping.

### *Surgical Procedure*

**Fig. 1:**
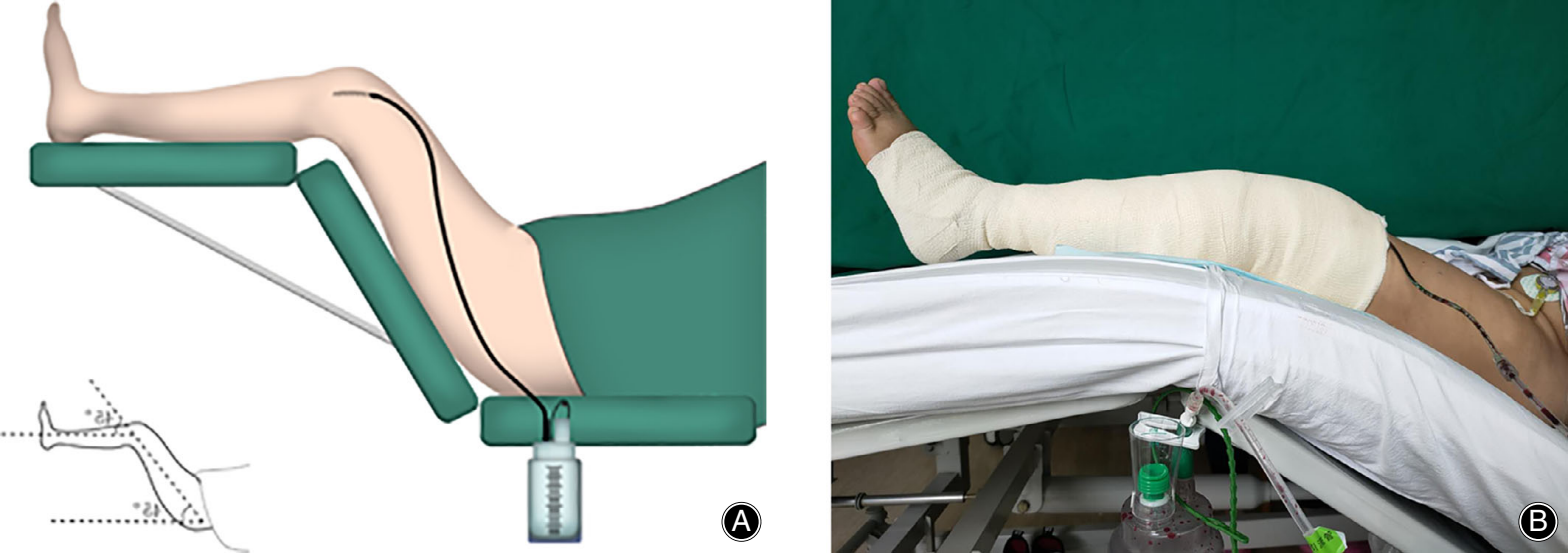
The position adopted in all patients in the tranexamic acid (TXA) group. The knee and hip were flexed at 45° in the TXA group

Any drug affecting coagulation was terminated 7 days before surgery. All operations were performed under spinal anesthesia through the medial parapatellar approach by the same surgical team specialized in TKA (10 years of experience working altogether), and the primary surgeon was one of the authors (D.C.). Synovial capsular and patellar tendon fat pads were excised. Osteophyte excision and soft tissue release were performed when necessary. A Press Fit Condyle (DePuy, Warsaw, USA) cemented prosthesis was used. The patella was reshaped to better match the shape of the femoral component trochlea in all cases. During the operation, a pneumatic tourniquet was applied at the proximal thigh with a pressure of 350 mmHg. Soft tissue balancing was achieved. The axial deformity was corrected in all cases. In each knee, one drainage tube was used, and it was connected to a high‐vacuum drain bottle (−600 mmHg, MEDINORM GmbH, Spiesen‐Elversberg, Germany), which was clamped for 4 h after the operation. In the TXA group, 500 mg of TXA mixed with 50 mL of physiological saline was injected into the knee joint before tourniquet release and after the closure of surgical wounds. Then, the affected knee and hip were flexed at 45° using a folding hospital bed after transporting the patients to the recovery ward; the position was retained for 4 h (Fig. 1). The patients in the control group had the same volume of physiological saline injected as a placebo, and the affected limb was fully extended. A high‐elasticity bandage was applied to compress the wound in all patients.

### *Postoperative Management*

The drainage tubes were removed at 24 h after the operation. The volume of postoperative blood loss in the vacuum drain bottle was recorded by the nurses. The patients performed ankle pumping exercises under the supervision of a physical therapist as soon as possible. Active isometric quadriceps exercises, extension–flexion motion, and initiative straight‐leg raising were encouraged after the operation. Patients could initiate ambulation with a walker after a satisfactory long‐leg radiograph examination confirmed a successful operation. All patients were given pharmacological thromboprophylaxis of low‐molecular‐weight heparin combined with mechanical thromboprophylaxis by ankle pumping, as mentioned above. Allogeneic transfusion was performed when hemoglobin levels were <7.0 g/dL or when hemoglobin was 7.0–9.0 g/dL combined with a poor mental status, palpitation, or pallor. No blood transfusion was performed for patients with hemoglobin levels >9.0 g/dL.

### *Outcome Measures*

#### *Postoperative Hemoglobin and Hematocrit*

The postoperative levels of hemoglobin were measured at four time points: 6 h after operation, and on the first, second, and third postoperative days. The hematocrit (Hct) values used in the following formula were measured preoperatively and at 24 h postoperatively. The calculated blood loss (CBL) was obtained using the formula reported by Nadler *et al*.^28^ and Gross^29^ as CBL = PBV × (hematocrit_baseline_ − hematocrit_day 3_)/hematocrit_average_. Higher hemoglobin levels indicate a higher capacity for oxygen transport and better postoperative outcomes^7^, ^30^. The changes in hemoglobin levels were the primary study endpoint.

#### *Blood Loss*

Wound drainage was the volume of blood drained over 24 h postoperatively minus the 50 mL injected with TXA. Hidden blood loss (HBL) was calculated by subtracting the visible blood loss (VBL) from the CBL at 24 h after the operation. Allogeneic blood transfusion was performed according to the blood transfusion protocol and calculated transfusion rate. Larger blood loss is associated with poorer postoperative outcomes^7^, ^30^. Patients who receive allogeneic blood transfusion are at risk of complications^7^.

#### *Knee Circumference*

Knee circumference (KC) indicates edema, and the changes in KC over time indicate swelling and its resolution. KC at the level of the superior patellar pole was measured at 6 days postoperatively, and the KCI was compared with the preoperative measurement. The KCI was measured using a measuring tape in the supine position, with the leg at 0°, at the level of the crease line.

#### *Range of Motion*

The ROM was assessed in the sitting position at 6 days postoperatively. KC represents edema and fluids trapped in the joint, while ROM indicates the presence of adhesions and inflammation, preventing the correct motion of the joint.

#### *Pain and Adverse Events*

The postoperative VAS pain score and adverse events (wound infection, pulmonary embolism, and deep vein thrombosis [DVT]) were recorded and analyzed.

### *Sample Size*

Power analysis and sample size estimation were performed using G*Power 3.1.7 (Franz Faul, Uni Kiel, Germany)^31^, ^32^, assuming: α (two‐tailed) = 0.05; level of β = 0.1 (90% power); and effect size = 1.1, which was determined based on the results of our preliminary experiment. This estimation resulted in a sample size of 40 patients per group, for a total of 80 patients. We added a 20% dropout rate for improper data collection. Thus, a sample size of 48 patients in each group was considered sufficient.

### *Statistical Analysis*

Data were analyzed using SPSS 19.0 (IBM, Armonk, NY, USA). Continuous variables, such as age, CBL, and Hb reduction, were tested for normal distribution using the Kolmogorov–Smirnov test. Normally distributed continuous data are expressed as means ± standard deviation and were analyzed using the Student's *t*‐test (intergroup comparisons) and repeated measure analysis of variance with the least significant difference post hoc test (intragroup comparisons). If the numerical variable had non‐normal distribution or unequal variance, the Wilcoxon Mann–Whitney *U*‐test was used. Categorical variables, such as gender and transfusion, are presented as frequencies and were analyzed using the χ^2^‐test or Fisher's exact test, as appropriate. *P*‐values <0.05 were considered statistically significant.

## Results

### *Characteristics of the Patients*

**Fig. 2:**
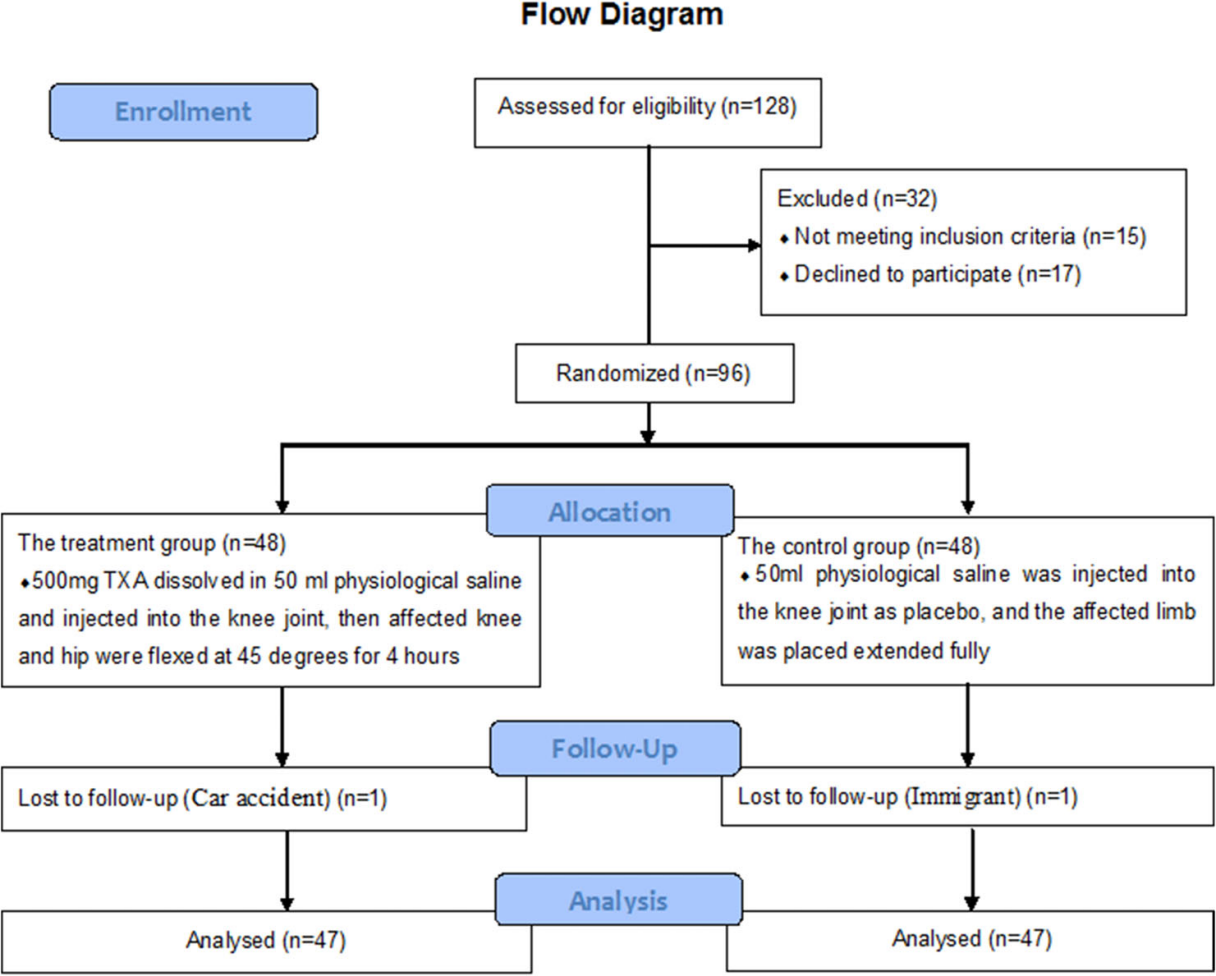
CONSORT patient flowchart. After screening based on the eligibility criteria, 128 patients were deemed eligible. Nineteen patients did not provide informed consent, and the remaining 96 patients participated in this randomized clinical trial. One patient in the tranexamic acid (TXA) group had a car accident 1 week after discharge. One patient in the control group emigrated to another city after leaving the hospital.

After screening based on the eligibility criteria, 128 patients were deemed eligible. Nineteen patients did not provide informed consent, and the remaining 96 patients participated in this randomized clinical trial. The primary diagnosis was osteoarthritis in all cases. One patient in the TXA group had a car accident 1 week after discharge. One patient in the control group emigrated to another city after leaving the hospital (Fig. 2).

The 94 remaining patients consisted of 24 men and 70 women. Patient ages ranged from 56 to 79 years (average, 64.8 years). A complete follow‐up was available for all patients. There were no significant differences in sex, age, affected side, body mass index, preoperative VAS pain score, preoperative hemoglobin, preoperative Hct, preoperative KC, and preoperative ROM (Table 1).

**TABLE 1:** Characteristics of the patients

Variable	TXA group (*n* = 47)	Control group (*n* = 47)	*P‐*value
Gender (male : female)	11:36	13:34	0.636
Age (years)	65.2 ± 6.2	64.4 ± 5.8	0.527
Affected side (left : right)	21:26	24:23	0.536
Body mass index (kg/m^2^)	26.0 ± 4.1	26.7 ± 3.9	0.421
Preoperative VAS score	7.2 ± 1.3	7.0 ± 1.2	0.668
Preoperative hemoglobin (g/dL)	13.1 ± 1.0	13.2 ± 1.1	0.621
Preoperative hematocrit (%)	39.1 ± 2.6	40.2 ± 3.1	0.077
Preoperative knee circumference (cm)	35.7 ± 3.4	36.0 ± 3.0	0.368
Preoperative range of motion (°)	93.8 ± 14.9	92.7 ± 11.3	0.680

TXA, tranexamic acid; VAS, visual analog scale.

### *Postoperative Hemoglobin and Hematocrit*

**Fig. 3:**
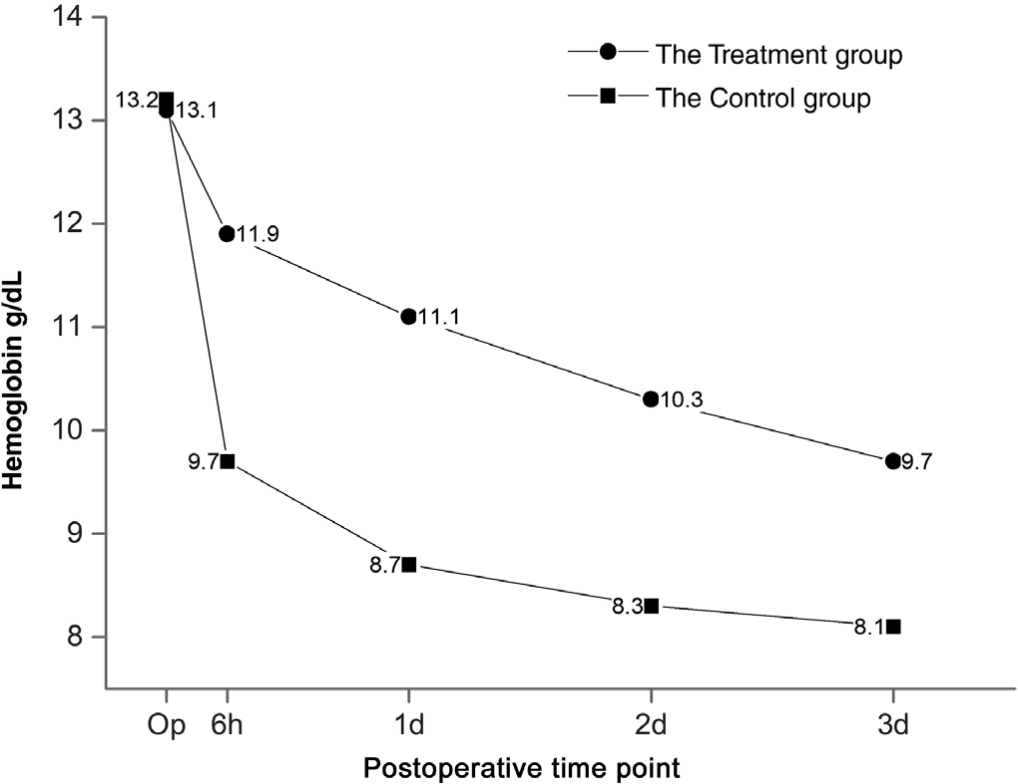
The trend of hemoglobin change during the perioperative period after total knee arthroplasty (TKA) in the tranexamic acid (TXA) and control groups. Hemoglobin levels were significantly higher in the TXA group (*P* < 0.05) at 6 h, 1 day, 2 days, and 3 days.

Postoperative characteristics are presented in Table 2. Postoperatively, hemoglobin levels were significantly higher in the TXA group (*P* < 0.05) at 6 h, 1 day, 2 days, and 3 days (Fig. 3). Hemoglobin reduction was significantly smaller, by 56%, in the TXA group (2.0 ± 0.9 *vs* 4.5 ± 0.7 g/dL, *P* < 0.05). The hematocrit reduction in the TXA group was significantly smaller than in the control group (*P* < 0.05).

**TABLE 2:** Blood loss

Variable	TXA group (*n* = 47)	Control group (*n* = 47)	*P‐*value
Hemoglobin (g/dL)			
6 h postoperation	11.9 ± 0.9	9.7 ± 0.8	<0.001	
1 day postoperation	11.1 ± 1.2	8.7 ± 1.0	<0.001	
2 days postoperation	10.3 ± 1.4	8.3 ± 0.9	<0.001	
3 days postoperation	9.7 ± 0.9	8.1 ± 0.8	<0.001	
Hemoglobin reduction (g/dL)	2.0 ± 0.9	4.5 ± 0.7	<0.001	
Hematocrit reduction (%)	6.61 ± 3.69	8.13 ± 3.9	<0.001	
Calculated blood loss (mL)	791 ± 212	1175 ± 273	<0.001	
Visible blood loss (mL)	382 ± 95	533 ± 111	<0.001	
Hidden blood loss (mL)	409 ± 125	642 ± 178	<0.001	
Transfusion	3 (6.4%)	13 (27.6%)	0.006	
Postoperative visual analog scale score	4.1 ± 1.1	4.8 ± 1.3	0.004	
Knee circumference increment (cm)	2.4 ± 0.9	3.2 ± 1.0	0.012	
Range of motion 6 days postoperation (°)	90.8 ± 6.2	87.6 ± 6.4	0.004	

TXA, tranexamic acid.

### *Blood Loss*

The CBL was smaller by 33% in the TXA group compared with the control group (791 ± 212 mL *vs* 1175 ± 273 mL, *P* < 0.001).

The mean visible blood loss in the TXA group was significantly less than in the control group (382 ± 95 mL *vs* 533 ± 111 mL, *P* < 0.001).

The hidden blood loss after TKA was significantly different between the two groups (409 ± 125 mL *vs* 642 ± 178 mL, *P* < 0.001). There was more hidden blood loss in the control group (Table 2).

Based on the transfusion criteria, 3 out of 47 (6.4%) patients in the TXA group and 13 out of 47 (27.6%) patients in the control group received blood transfusions (*P* = 0.006). One transfused patient had a hemoglobin level <8.5 g/dL that was associated with significantly declined mental status and pallor in the control group, and the other 15 transfused patients had hemoglobin <7.0 g/L.

### *Knee Circumference*

At 6 days after TKA, the KCI was smaller by 25% in the TXA group than in the control group (2.4 ± 0.9 cm *vs* 3.2 ± 1.0 cm, *P* = 0.012).

### *Range of Motion*

At 6 days after the operation, the ROM was significantly larger by 3° in the TXA group compared with controls (90.8° ± 6.2° *vs* 87.6° ± 6.4°, *P* = 0.004).

### *Pain*

The VAS pain scores were lower by 15% in the TXA group compared with controls (*P* = 0.004).

### *Adverse Events*

There was no deep venous thrombosis (DVT), wound infection, or other adverse event in either group. In the control group, 2 patients had a fever after blood transfusion.

## Discussion

Because of extensive soft tissue release and bone cutting, excessive blood loss is still a problem during TKA. Blood transfusion, which can lead to a great burden to both the patients' families and society, is the main method to deal with this problem at present. In addition, there are several risks associated with transfusion, including transfusion reactions, intravascular hemolysis, infection, renal failure, and fluid overload^13^, ^33^, ^34^. This study examined TXA administration and joint flexion for hemostasis in patients undergoing TKA. The results suggest that the intra‐articular injection of TXA combined with knee and hip flexion at 45° can effectively attenuate CBL by 32.7% and hemoglobin reduction by 55.6% during primary TKA, without an additional adverse event.

Over the past few decades, different methods of limb positioning after TKA have been proposed to reduce blood loss caused by TKA and to prevent the need for blood transfusion^21^, ^22^, ^23^, ^35^, ^36^, ^37^. The extent of knee flexion ranges from 30° to 120°, and hip flexion ranges from 30° to 90°. It has been hypothesized that knee flexion can increase local muscle tension around the knee and decrease venous return to the knee joint cavity, thus reducing VBL^26^. In addition, popliteal vein entrapment can be relieved, and the affected limb's venous blood backflow to the heart can be accelerated, thus reducing HBL and reducing swelling more quickly^23^, ^38^. Because maintaining knee flexion after TKA is painful, 45° of flexion was selected in the present study. This method was also used by Liu *et al*.^22^, and the results showed that 45° of flexion at the knee and hip was an effective and simple method to reduce blood loss by 16.8%, supporting the results of the present study.

Intravenous TXA can reduce blood loss by nearly 30% and the need for a blood transfusion by 53%^4^, ^39^. Roy *et al*.^40^ reported that intra‐articular administration of 500 mg of TXA could reduce drain output by 52% at 48 h after the operation and decrease hemoglobin levels by 36%. Even so, the effect of TXA in reducing blood loss and transfusion in patients undergoing a TKA operation remains controversial^41^, ^42^. In the present study, we referred to and improved the methods described by Roy *et al*.^40^ Although the TXA dose injected into the knee joint was the same, the VBL (382 mL in the TXA group and 533 mL in the control group) was lower than that reported by Roy *et al*.^40^ (401 mL in the TXA group and 870 mL in the control group). Probable reasons for this discrepancy include the following. First, the volume of saline that we injected into the knee joint was 50 mL and not 5 mL, which means that the mixed preparations occupied more space in the knee joint cavity, probably distributing the TXA more evenly. Then, a relatively small amount of bleeding can lead to a full joint cavity after the operation, which can enhance the hematoma filling effect. Second, the drainage clamp time in the present study was 4 h and not 1 h, as in Roy *et al*.^40^ The prolonged clamping time of the drainage tubes resulted in a longer tissue contact time, which could enhance the effect of TXA and result in more effective control of postoperative blood loss. Finally, when the knee is flexed, the angulation of popliteal vessels and the relaxation of muscles slow down venous blood flow, which can provide appropriate conditions for blood clotting response. In the present study, a significant difference in hemoglobin reduction (2.0 g/dL in the TXA group and 4.5 g/dL in the control group) was observed between the two groups. As for the transfusion rate, the proportion of patients in the TXA group who needed a transfusion was lower (6.4%) than that in a retrospective cohort study of 872,416 patients who received TXA in the United States in 2014 (7.7%)^43^. This endpoint may be a result of the combined techniques resulting in a superimposed hemostasis effect of TXA application and knee flexion.

Previous reports demonstrated a dose‐dependent effect of TXA on decreasing blood loss^44^, but an excessive dose of TXA may result in potential adverse drug reactions. Alshryda *et al*.^45^ compared the blood loss following TKA between the TXA 1000 mg group and the control group and found that 1000 mg of TXA intra‐articularly can reduce total blood loss by 18.4% and attenuate hemoglobin reduction by 10%^45^. In the present study, the 500 mg of TXA administered intra‐articularly combined with limb flexion and clamped drainage for 4 h showed a better effect on hemoglobin reduction (2.0 g/dL) than did the 1000 mg of TXA administered intra‐articularly (3.07 g/dL)^45^, without increasing the risk of an adverse event.

Hidden blood loss is the primary cause of soft tissue swelling around the knee joint after TKA^46^, ^47^. In the present study, HBL was lower in the TXA group than in the control group (409 mL *vs* 642 mL), resulting in decreased limb swelling (KCI, 2.4 cm *vs* 3.2 cm, respectively, *P* < 0.05) and a better functional endpoint (ROM 6 days postoperative, 90.8° *vs* 87.6°) in the early postoperative period.

The transfusion rate is a general parameter for evaluating the hemostatic effect that has been used in previous literature, but it is an unreasonable parameter because it is affected by preoperative hemoglobin levels and the local blood transfusion protocol^30^.

A possible limitation of this study is the lack of comparison with patients who only had limb flexion and who only received TXA administration. Therefore, we cannot clarify how much of the positive contribution to the hemostatic effect was made by limb flexion and how much was by intra‐articular TXA administration. More studies using possible combinations of different blood‐saving techniques are needed in the future to provide better blood loss management. Despite this limitation, the patients enrolled in our study all had knee OA, not rheumatoid arthritis or traumatic arthritis, and identical knee prostheses were used in the operations. These parameters should reduce experimental error.

In conclusion, the intra‐articular administration of 500 mg of TXA combined with knee and hip flexion at 45° is an effective method for reducing postoperative blood loss, by 32.7%, and hemoglobin reduction, by 55.6%, in primary TKA, without increasing operation‐related adverse event.

### *Authorship declaration*

All authors listed meet the authorship criteria according to the latest guidelines of the International Committee of Medical Journal Editors, and all authors are in agreement with the manuscript.

## References

[1] Gelber AC . In the clinic. Osteoarthritis. Ann Intern Med, 2014, 161: ITC1‐16. 10.7326/0003-4819-161-1-201407010-01001 24979462

[2] Cross M , Smith E , Hoy D . The global burden of hip and knee osteoarthritis: estimates from the global burden of disease 2010 study. Ann Rheum Dis, 2014, 73: 1323–1330. 24553908 10.1136/annrheumdis-2013-204763

[3] Zhang W , Moskowitz RW , Nuki G , *et al* OARSI recommendations for the management of hip and knee osteoarthritis, Part II: OARSI evidence‐based, expert consensus guidelines. Osteoarthritis Cartilage, 2008, 16: 137–162. 18279766 10.1016/j.joca.2007.12.013

[4] Good L , Peterson E , Lisander B . Tranexamic acid decreases external blood loss but not hidden blood loss in total knee replacement. Br J Anaesth, 2003, 90: 596–599. 12697586 10.1093/bja/aeg111

[5] Pawaskar A , Salunke AA , Kekatpure A , *et al* Do autologous blood transfusion systems reduce allogeneic blood transfusion in total knee arthroplasty? Knee Surg Sports Traumatol Arthrosc, 2017, 25: 2957–2966. 27085359 10.1007/s00167-016-4116-z

[6] Si HB , Yang TM , Zeng Y , Shen B . No clear benefit or drawback to the use of closed drainage after primary total knee arthroplasty: a systematic review and meta‐analysis. BMC Musculoskelet Disord, 2016, 17: 183. 27118129 10.1186/s12891-016-1039-2 PMC4845483

[7] Liu D , Dan M , Martinez Martos S , Beller E . Blood management strategies in total knee arthroplasty. Knee Surg Relat Res, 2016, 28: 179–187. 27595070 10.5792/ksrr.2016.28.3.179 PMC5009041

[8] Hebert PC , Wells G , Tweeddale M , *et al* Does transfusion practice affect mortality in critically ill patients? Transfusion Requirements in Critical Care (TRICC) Investigators and the Canadian Critical Care Trials Group. Am J Respir Crit Care Med, 1997, 155: 1618–1623. 9154866 10.1164/ajrccm.155.5.9154866

[9] Bernard AC , Davenport DL , Chang PK , Vaughan TB , Zwischenberger JB . Intraoperative transfusion of 1 U to 2 U packed red blood cells is associated with increased 30‐day mortality, surgical‐site infection, pneumonia, and sepsis in general surgery patients. J Am Coll Surg, 2009, 208: 931–937–937. 19476865 10.1016/j.jamcollsurg.2008.11.019

[10] Bower WF , Jin L , Underwood MJ , Lam YH , Lai PB . Peri‐operative blood transfusion increases length of hospital stay and number of postoperative complications in non‐cardiac surgical patients. Hong Kong Med J, 2010, 16: 116–120. 20354245

[11] Newman ET , Watters TS , Lewis JS , *et al* Impact of perioperative allogeneic and autologous blood transfusion on acute wound infection following total knee and total hip arthroplasty. J Bone Joint Surg Am, 2014, 96: 279–284. 24553883 10.2106/JBJS.L.01041

[12] Innerhofer P , Klingler A , Klimmer C , Fries D , Nussbaumer W . Risk for postoperative infection after transfusion of white blood cell‐filtered allogeneic or autologous blood components in orthopedic patients undergoing primary arthroplasty. Transfusion, 2005, 45: 103–110. 15647025 10.1111/j.1537-2995.2005.04149.x

[13] Bong MR , Patel V , Chang E , Issack PS , Hebert R , Di Cesare PE . Risks associated with blood transfusion after total knee arthroplasty. J Arthroplasty, 2004, 19: 281–287. 15067638 10.1016/j.arth.2003.10.013

[14] Kullenberg B , Ylipaa S , Soderlund K , Resch S . Postoperative cryotherapy after total knee arthroplasty: a prospective study of 86 patients. J Arthroplasty, 2006, 21: 1175–1179. 17162178 10.1016/j.arth.2006.02.159

[15] Holt JB , Miller BJ , Callaghan JJ , Clark CR , Willenborg MD , Noiseux NO . Minimizing blood transfusion in total hip and knee arthroplasty through a multimodal approach. J Arthroplasty, 2016, 31: 378–382. 26391927 10.1016/j.arth.2015.08.025

[16] Krebs V , Hozack WJ , Callaghan JJ , Bohannon Mason J , Mont M , Parvizi J . Eliminating transfusion in primary joint arthroplasty‐an achievable goal. J Arthroplasty, 2014, 29: 1511. 25085212 10.1016/j.arth.2014.07.014

[17] Benoni G , Carlsson A , Petersson C , Fredin H . Does tranexamic acid reduce blood loss in knee arthroplasty? Am J Knee Surg, 1995, 8: 88–92. 7552611

[18] Wang G , Wang D , Wang B , Lin Y , Sun S . Efficacy and safety evaluation of intra‐articular injection of tranexamic acid in total knee arthroplasty operation with temporarily drainage close. Int J Clin Exp Med, 2015, 8: 14328–14334. 26550418 PMC4613103

[19] Hamlin BR , DiGioia AM , Plakseychuk AY , Levison TJ . Topical versus intravenous tranexamic acid in total knee arthroplasty. J Arthroplasty, 2015, 30: 384–386. 25458092 10.1016/j.arth.2014.10.007

[20] Bagsby DT , Hur J . Effect of intra‐articular injection of tranexamic acid on postoperative hemoglobin in total hip arthroplasty. Orthopedics, 2014, 37: e557–e562. 24972437 10.3928/01477447-20140528-56

[21] Yang Y , Yong‐Ming L , Pei‐jian D , Jia L , Ying‐ze Z . Leg position influences early blood loss and functional recovery following total knee arthroplasty: a randomized study. Int J Surg, 2015, 23: 82–86. 26407829 10.1016/j.ijsu.2015.09.053

[22] Liu J , Li YM , Cao JG , Wang L . Effects of knee position on blood loss following total knee arthroplasty: a randomized, controlled study. J Orthop Surg Res, 2015, 10: 69. 25982235 10.1186/s13018-015-0213-9 PMC4443627

[23] Napier RJ , Bennett D , McConway J , *et al* The influence of immediate knee flexion on blood loss and other parameters following total knee replacement. Bone Joint J, 2014, 96‐b: 201–209. 10.1302/0301-620X.96B2.32787 24493185

[24] Fillingham YA , Ramkumar DB , Jevsevar DS , *et al* The efficacy of tranexamic acid in total knee arthroplasty: a network meta‐analysis. J Arthroplasty, 2018, 33: 3090–3098. e1. 29805106 10.1016/j.arth.2018.04.043

[25] Guo P , He Z , Wang Y , *et al* Efficacy and safety of oral tranexamic acid in total knee arthroplasty: a systematic review and meta‐analysis. Medicine (Baltimore), 2018, 97: e0587. 29718858 10.1097/MD.0000000000010587 PMC6393150

[26] Faldini C , Traina F , De Fine M , Pedrini M , Sambri A . Post‐operative limb position can influence blood loss and range of motion after total knee arthroplasty: a systematic review. Knee Surg Sports Traumatol Arthrosc, 2015, 23: 852–859. 24682489 10.1007/s00167-013-2732-4

[27] Kellgren JH , Lawrence JS . Radiological assessment of rheumatoid arthritis. Ann Rheum Dis, 1957, 16: 485–493. 13498603 10.1136/ard.16.4.485 PMC1006994

[28] Nadler SB , Hidalgo JH , Bloch T . Prediction of blood volume in normal human adults. Surgery, 1962, 51: 224–232. 21936146

[29] Gross JB . Estimating allowable blood loss: corrected for dilution. Anesthesiology, 1983, 58: 277–280. 6829965 10.1097/00000542-198303000-00016

[30] Maempel JF , Wickramasinghe NR , Clement ND , Brenkel IJ , Walmsley PJ . The pre‐operative levels of haemoglobin in the blood can be used to predict the risk of allogenic blood transfusion after total knee arthroplasty. Bone Joint J, 2016, 98‐B: 490–497. 10.1302/0301-620X.98B4.36245 27037431

[31] Faul F , Erdfelder E , Lang AG , Buchner A . G*Power 3: a flexible statistical power analysis program for the social, behavioral, and biomedical sciences. Behav Res Methods, 2007, 39: 175–191. 17695343 10.3758/bf03193146

[32] Faul F , Erdfelder E , Buchner A , Lang AG . Statistical power analyses using G*Power 3.1: tests for correlation and regression analyses. Behav Res Methods, 2009, 41: 1149–1160. 19897823 10.3758/BRM.41.4.1149

[33] Innerhofer P , Walleczek C , Luz G , *et al* Transfusion of buffy coat‐depleted blood components and risk of postoperative infection in orthopedic patients. Transfusion, 1999, 39: 625–632. 10378843 10.1046/j.1537-2995.1999.39060625.x

[34] Gillette BP , Maradit Kremers H , Duncan CM , *et al* Economic impact of tranexamic acid in healthy patients undergoing primary total hip and knee arthroplasty. J Arthroplasty, 2013, 28: 137–139. 23886409 10.1016/j.arth.2013.04.054

[35] Li B , Wen Y , Liu D , Tian L . The effect of knee position on blood loss and range of motion following total knee arthroplasty. Knee Surg Sports Traumatol Arthrosc, 2012, 20: 594–599. 21811855 10.1007/s00167-011-1628-4

[36] Madarevic T , Tudor A , Sestan B , *et al* Postoperative blood loss management in total knee arthroplasty: a comparison of four different methods. Knee Surg Sports Traumatol Arthrosc, 2011, 19: 955–959. 21076814 10.1007/s00167-010-1309-8

[37] Ma T , Khan RJ , Carey Smith R , Nivbrant B , Wood DJ . Effect of flexion/extension splintage post total knee arthroplasty on blood loss and range of motion – a randomised controlled trial. Knee, 2008, 15: 15–19. 17997100 10.1016/j.knee.2007.09.004

[38] Ong SM , Taylor GJ . Can knee position save blood following total knee replacement? Knee, 2003, 10: 81–85. 12649032 10.1016/s0968-0160(02)00076-5

[39] Molloy DO , Archbold HA , Ogonda L , McConway J , Wilson RK , Beverland DE . Comparison of topical fibrin spray and tranexamic acid on blood loss after total knee replacement: a prospective, randomised controlled trial. J Bone Joint Surg Br, 2007, 89: 306–309. 17356139 10.1302/0301-620X.89B3.17565

[40] Roy SP , Tanki UF , Dutta A , Jain SK , Nagi ON . Efficacy of intra‐articular tranexamic acid in blood loss reduction following primary unilateral total knee arthroplasty. Knee Surg Sports Traumatol Arthrosc, 2012, 20: 2494–2501. 22419263 10.1007/s00167-012-1942-5

[41] Chen X , Zhu X , Yang S , Lin W , Wang L . Tranexamic acid treatment decreases hidden blood loss in total knee arthroplasty. Am J Ther, 2016, 23: e1397–e1405. 25768379 10.1097/MJT.0000000000000230

[42] Chen X , Cao X , Yang C , Guo K , Zhu Q , Zhu J . Effectiveness and safety of fixed‐dose tranexamic acid in simultaneous bilateral total knee arthroplasty: a randomized double‐blind controlled trial. J Arthroplasty, 2016, 31: 2471–2475. 27167769 10.1016/j.arth.2016.04.003

[43] Poeran J , Rasul R , Suzuki S , *et al* Tranexamic acid use and postoperative outcomes in patients undergoing total hip or knee arthroplasty in the United States: retrospective analysis of effectiveness and safety. BMJ, 2014, 349: g4829. 25116268 10.1136/bmj.g4829 PMC4130961

[44] Jang B , Kao M , Bohm MT , Harris IA , Chen DB , MacDessi SJ . Intra‐articular injection of tranexamic acid to reduce blood loss after total knee arthroplasty. J Orthop Surg (Hong Kong), 2014, 22: 146–149. 25163943 10.1177/230949901402200205

[45] Alshryda S , Mason J , Sarda P , *et al* Topical (intra‐articular) tranexamic acid reduces blood loss and transfusion rates following total hip replacement: a randomized controlled trial (TRANX‐H). J Bone Joint Surg Am, 2013, 95: 1969–1974. 24196467 10.2106/JBJS.L.00908

[46] Schroder D , Passler HH . Combination of cold and compression after knee surgery. A prospective randomized study. Knee Surg Sports Traumatol Arthrosc, 1994, 2: 158–165. 7584198 10.1007/BF01467918

[47] Ishida K , Tsumura N , Kitagawa A , *et al* Intra‐articular injection of tranexamic acid reduces not only blood loss but also knee joint swelling after total knee arthroplasty. Int Orthop, 2011, 35: 1639–1645. 21253725 10.1007/s00264-010-1205-3 PMC3193960

